# Inhibition of insulin receptor isoform-A signalling restores sensitivity to gefitinib in previously *de novo* resistant colon cancer cells

**DOI:** 10.1038/sj.bjc.6603237

**Published:** 2006-07-04

**Authors:** H E Jones, J M W Gee, D Barrow, D Tonge, B Holloway, R I Nicholson

**Affiliations:** 1 Tenovus Centre for Cancer Research, Welsh School of Pharmacy, Cardiff University, Cardiff, UK; 2 AstraZeneca Pharmaceuticals, Macclesfield, Cheshire, UK

**Keywords:** EGFR, Insulin receptor-isoform A, gefitinib, resistance

## Abstract

Resistance to antiepidermal growth factor (EGFR) strategies is an emerging clinical problem. Using human colorectal cancer (CRC) cells, we evaluated the involvement of the insulin receptor isoform-A (InsR-A) in *de novo* resistance to gefitinib, an EGFR tyrosine kinase inhibitor. Challenging the EGFR positive LoVo cells with gefitinib (1 *μ*M) resulted in a small (∼18%) inhibition of cell growth and although a modest reduction in phospho (p)EGFR Tyr845 was seen, pEGFR at residues -Tyr1068 and -Tyr1173 were unchanged. LoVo cells produced unprocessed pro-IGF-1R protein, substantial levels of IGF-II mRNA and mature InsR protein, consisting mainly of the InsR-A isoform. Insulin and IGF-II promoted cell growth and pEGFR Tyr845, Tyr1068 and Tyr1173 activity and conversely, the insulin-like growth factor-1 receptor (IGF-1R)/InsR inhibitor ABDP (1 *μ*M) inhibited growth and reduced pEGFR activity at all three tyrosine residues. pInsR and pAkt levels were increased after gefitinib treatment. Blocking of pInsR with ABDP enabled gefitinib to markedly reduce pEGFR Tyr845, Tyr1068 and Tyr1173. Short-term gefitinib/ABDP dual treatment was more effective than either agent alone and chronic exposure to this combination resulted in total cell loss after 9 weeks, preventing acquisition of resistance to ABDP. LoVo cells with acquired resistance to ABDP were acutely sensitive to gefitinib. We concluded that InsR-A reduces sensitivity to gefitinib in LoVo CRC cells, thus its co-targeting alongside EGFR can improve the anti-tumour effect of gefitinib.

Colorectal cancer (CRC) is one of the most commonly occurring human malignancies (Parkin, 2001) and in advanced CRC, the epidermal growth factor receptor (EGFR) and/or its ligands are frequently overexpressed and have been implicated in increased risk of metastasis and hence poor prognosis (reviewed by Spano *et al*, 2005) and additionally, increased EGFR expression has also been associated with chemo-refractory disease (Saltz *et al*, 2001). The EGFR therefore, represents a promising therapeutic target in CRC (Spano *et al*, 2005). The EGFR is a membrane glycoprotein consisting of a ligand binding region, a transmembrane segment and an intracellular portion, the latter containing a tyrosine kinase domain which is upstream of additional autophosphorylation tyrosine sites within the carboxy terminus of the receptor (Gullick, 2001). Association of the ligand induces receptor dimerisation and activates the tyrosine kinase domains of each receptor which results in the transautophosphorylation of the tyrosine sites on the other EGFR molecule, activating signalling transduction cascades which regulate both normal and tumourigenic cellular processes (Gullick, 2001). Among current strategies for targeting the EGFR are small molecule inhibitors, including gefitinib (Iressa™), which act by specifically inhibiting the activity of the EGFR tyrosine kinase (Ciardiello and Tortora, 2001).

Both *in vitro* and *in vivo* studies indicated that gefitinib had antitumour activity as a monotherapy in some (Ciardiello *et al*, 2000, 2001) but not all CRC cell lines investigated (Williams *et al*, 2002). Other studies reported that gefitinib could have a synergistic interaction with chemotherapeutic agents (Ciardiello *et al*, 2000; Xu *et al*, 2003) and radiotherapy in xenograft models for CRC (Williams *et al*, 2002). Disappointingly, however, phase I/II clinical studies in patients with advanced CRC indicated that gefitinib had negligible single agent activity (Goss *et al*, 2002; Rothenberg *et al*, 2004) but the inhibitor was seen to demonstrate modest activity when used in combination with 5-fluororacil, leucovorin and irinotecan (Veronese *et al*, 2005) or FOLFOX-4 (Cho *et al*, 2005; Kuo *et al*, 2005). Additionally, clinical data in other cancer types has also indicated the existence of *de novo* and acquired resistance to gefitinib (Ranson *et al*, 2002; Schiller, 2002; Kelly and Averbuch, 2004) and interestingly, a clear link between EGFR levels and predicted response to EGFR-targeted agents including gefitinib has not been observed (Arteaga, 2002; Ranson *et al*, 2002; Saltz *et al*, 2004). Recent evidence has implicated signalling via the type II receptor tyrosine kinase (RTK) family member the insulin-like growth factor-1 receptor (IGF-1R) and resistance to various anti-EGFR therapies (Liu *et al*, 2001; Chakravarti *et al*, 2002; Jones *et al*, 2004). Another member of the type II RTK family however, is the insulin receptor (InsR) which shows a high degree of homology with the IGF-1R (De Meyts and Wittaker, 2002) and has been reported to play a role in cancer development and progression (Denley *et al*, 2003). The InsR occurs in two isoforms which are produced by the tissue specific alternative splicing of exon 11, insulin receptor isoform-A (InsR-A) (Ex11−) and InsR-B (Ex11+) (Mosthaf *et al*, 1990). Both isoforms can bind insulin with high affinity (Yamaguchi *et al*, 1993), but InsR-A can also bind IGF-II with high affinity (Frasca *et al*, 1999). Furthermore, stimulation of the InsR-A by insulin and IGF-II can promote cancer cell mitogenesis and survival (reviewed by Denley *et al*, 2003). In addition, several clinical cancer types including CRC, have been shown to preferentially express InsR-A (Frasca *et al*, 1999; Vella *et al*, 2002).

In this study, we demonstrate for the first time that LoVo CRC cells, known to lack functional IGF-1R (Lehmann *et al*, 1998), not only express mainly InsR-A *vs* InsR-B, providing an opportunity to study InsR-A without molecular manipulation , but furthermore, the InsR-A can modulate EGFR phosphorylation in the presence of gefitinib and hence, contribute to the lack of sensitivity to this inhibitor shown by the EGFR positive LoVo cells. Consequently, we observed that gefitinib sensitivity could be restored by minimising InsR activity, which translated into a combination treatment of gefitinib with an IGF-1R/InsR inhibitor being considerably more efficacious than either inhibitor given as a single agent. Additionally, gefitinib is extremely effective in cells which have acquired resistance to the InsR inhibitor, an observation which may have important ramifications for the scheduling of agents that target the EGFR. The study clearly demonstrates the need to elucidate the potential mechanisms underpinning resistance to anti-EGFR agents such as gefitinib in order to rationally design combination drug regimes to improve drug efficacy and maximise antitumour effects. In addition, usually overshadowed by its more well-known family member, the IGF-1R, this work also highlights the growing importance of the InsR-A as a future therapeutic anticancer target.

## MATERIALS AND METHODS

### Cell culture

LoVo CRC cells (gifted from AstraZeneca Pharmaceuticals, Macclesfield, Cheshire, UK) were routinely cultured in phenol red Dulbecco's modified Eagle's Medium (DMEM), supplemented with 10% fetal calf serum (FCS) plus antibiotics. LoVo-ABDP-R cells were routinely maintained in phenol red DCCM-1 medium (Biological Industries, Cumbernauld, UK) containing 0.5% FCS and 1 *μ*M ABDP. Both routine and experimental medium was replaced every 4 days and the cultures were maintained at 37°C in a humidified 5% CO_2_ atmosphere.

### Long-term growth studies

Gefitinib and the IGF-1R/InsR tyrosine kinase inhibitor ABDP were gifts from AstraZeneca. LoVo cells were continuously exposed to 1 *μ*M gefitinib, 1 *μ*M ABDP or gefitinib and ABDP in combination, in phenol red DCCM-1 supplemented with 0.5% FCS. As gefitinib had little effect on the growth of the LoVo cells, both control and gefitinib treated cultures were passaged weekly at a seeding ratio of 1 : 10. Initially, ABDP alone dramatically reduced (80%) LoVo cell numbers and during the following 2 months, the surviving cells were passaged approximately every 2–3 weeks at a seeding ratio of 1 : 2. Over the following 2 months the cells were passaged once every 2 weeks at a seeding ratio of 1 : 4. A stable growth rate was reached after a total of 5 months with routine maintenance of the ABDP-resistant variant (LoVo-ABDP-R) involving passage every 7 days with a seeding ratio of 1 : 10 of the confluent cell number. The gefitinib and ABDP combination treatment also reduced LoVo cell number and were passaged twice during 2 months at a seeding ratio of 1 : 2, however, during the following month, the cell number declined below the seeding density and could not be maintained any further.

### Cell growth analysis

The LoVo and LoVo-ABDP-R cells were seeded into 24-well plates in routine cell medium at a density of 6 × 10^4^ cells well^−1^. After 24 h, the cells were washed with phosphate buffered saline (PBS) and the experimental treatments were added as detailed below. LoVo cells were cultured in phenol red DCCM-1 containing (a) various concentrations of FCS (0–10%) in the absence and presence of 1 *μ*M gefitinib, or (b) EGF, IGF-II (both at 10 ng ml^−1^) and insulin (10 *μ*g ml^−1^) or (c) various concentrations of the InsR/IGF-1R inhibitor ABDP (0–10 *μ*M) in the absence or presence of 1 *μ*M gefitinib with 0.5% FCS. LoVo-ABDP-R cells were challenged with EGF (10 ng ml^−1^) in the absence and presence of 1 *μ*M gefitinib in phenol red DCCM-1 with 1 *μ*M ABDP. Cell population growth was evaluated after 7 days by Coulter (Luton, UK) counting analysis. Each experiment was also performed in triplicate.

### Western blotting analysis

LoVo and LoVo-ABDP-R cells were seeded into 60 mm dishes in routine medium at a density of 7.5 × 10^5^ cells dish^−1^. After 24 h, the experimental treatment regimes were as follows: (a) LoVo cells were incubated in DCCM-1 with 0.5% FCS for 7 days in the absence or presence of either 1 *μ*M gefitinib or 1 *μ*M ABDP or (b) growth in routine culture medium until 70% confluent, serum starved in DCCM-1 for 24 h and subsequently challenged with insulin and IGF-II for 5 min or EGF, insulin and IGF-II with and without 1 *μ*M ABDP for 5 min. Cells exposed to ABDP were preincubated with this inhibitor for 6 h or (c) cells were cultured in the presence of 1 *μ*M ABDP for 4 days before the addition of 1 *μ*M gefitinib. LoVo-ABDP-R cells were grown in the absence and presence of 1 *μ*M gefitinib in phenol red DCCM-1 with 1 *μ*M ABDP for 7 days or challenged with insulin (10 *μ*g ml^−1^) for 5 min.

Cell monolayers were harvested and protein samples were electrophoresed, electroblotted onto nitrocellulose membranes and blocking solution utilised exactly as described previously (Jones *et al*, 2004), before incubation with a variety of antibodies at 1 : 1000 dilution unless stated otherwise for 3 h at room temperature. Antibodies used were total EGFR (1 : 4000) (1005, Santa Cruz, Biotechnology Inc. CA, USA), pEGFR Tyr845, pEGFR Tyr1068, total Akt, pAKT Ser473, total ERK1/2, pERK1/2 Thr202/Tyr204 and pIGF-1R/InsR Tyr1131/1146 (all from Cell Signaling, Beverly, MA, USA), pEGFR Tyr1173 (Upstate Biotechnology UK, Buckingham, UK) and total IGF-1R (N-20) and InsR (N-20) (both from Santa Cruz). The membranes were further incubated with the appropriate secondary IgG horse radish peroxidase labelled antibody and proteins were visualised by chemiluminescence. The resulting bands were analysed by scanning densitometry and normalised to *β*-actin.

### RT–PCR

RNA was harvested from the cells using an RNA Isolator kit (Sigma Chemical Co., Dorset, UK) according to the manufacturer's instructions. Total RNA (1 *μ*g) was reverse transcribed and the resulting cDNA was amplified using specific primer sets for TGF-*α* (5′ CCACACTCAGTTCTGCTTCC and 3′ TCTTTATTGATCTGCCACAGTC), insulin (5′ TCACACCTGGTGGAAGCTC and 3′ ACAATGCCACGCTTCTGC) and IGF-II (5′ TGGGAATCCCAATGGGGAAG and 3′ CTTGCCCACGGGGTATCT). Pancreatic cDNA (BD Biosciences, Erembodegem, Belgium) was utilised as a control for insulin. In parallel, *β*-actin c-DNA (5′ GGAGCAATGATCTTGATCTT and 3′ CCTTCCTGGGCATGGAGTCCT) was amplified in replicate samples as an internal control. PCR amplification consisted of 1 min at 94°C, 30 s at 55°C and 1 min at 72°C for 30, 35 and 27 cycles for TGF-*α*, insulin, IGF-II and *β*-actin, respectively. The resulting amplified c-DNA fragments were resolved on agarose gels stained with ethidium bromide, analysed by scanning densitometry and normalised relative to *β*-actin. InsR isoform detection was determined using primers as described by Sciacca *et al* (1999), which spanned nucleotides 2229–2250 (5′ AACCAGAGTGAGTATGAGGAT 3′) and 2844–2865 (5′ CCGTTCCAGAGCGAAGTGCTT 3′) of the human insulin receptor. PCR amplification was carried out for 35 cycles using the conditions detailed above. The PCR products were resolved on 15% polyacrylamide gels and fragments of 600 and 636 bp representing InsR-A Ex−11 and InsR-B Ex+11 were detected by ethidium bromide staining, scanned and normalised to *β*-actin as described previously. In order to verify that the larger cDNA fragment was InsR-B, the RT–PCR products were subjected to digestion with the restriction enzyme *Ban*I. Only cDNA containing exon 11, the restriction site for *Ban*I, was digested, leaving InsR-A Ex-11 intact.

### Statistical analysis

Overall differences between control and treatment groups were examined by means of a Kruskall–Wallis test. Direct comparisons between control and treatment effects were assessed using a two-sided Mann–Whitney test. Significance was determined at the *P*<0.05 level.

## RESULTS

### Effect of gefitinib on cell growth and EGFR phosphorylation status

**Figure 1:**
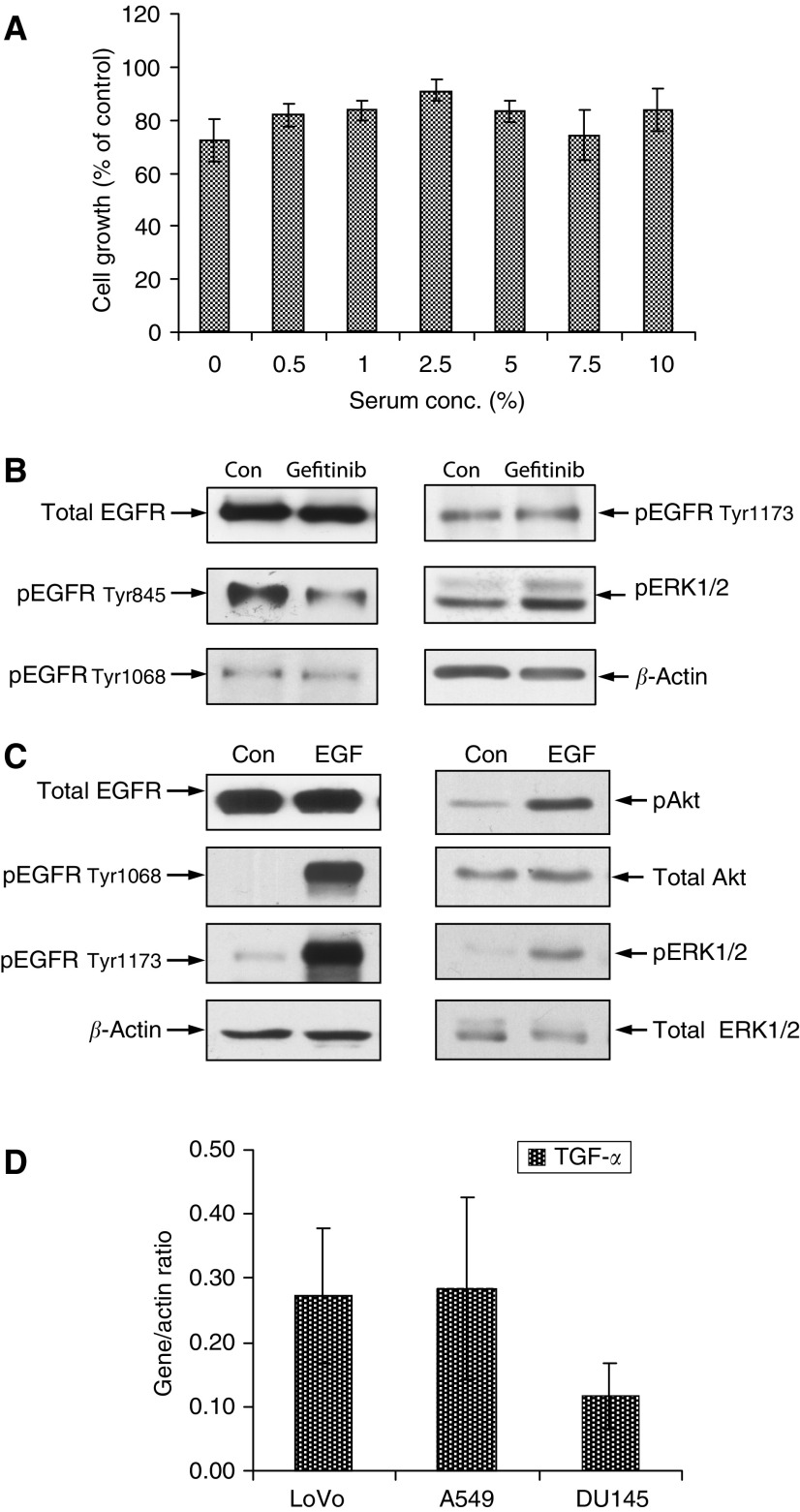
Response to gefitinib and basal EGFR expression and phosphorylation status. (**A**) LoVo cells were grown in DCCM-1 containing varying serum concentrations (0–10%) in the absence and presence of 1 *μ*M gefitinib. Data are mean values of three independent experiments (every point in each individual experiment also being evaluated in triplicate) and error bars represent 95% CIs. Significant differences were assessed at the *P*<0.05 level between the growth inhibition value obtained in the presence of 10% serum and gefitinib and the growth inhibitory values seen with each of the other serum concentrations in the presence of gefitinib. (**B**) Cells were grown in DCCM-1 with 0.5% serum in the absence and presence of 1 *μ*M gefitinib for 7 days. Protein (75 *μ*g) of cell lysate was electrophoresed by SDS–PAGE (7.5%) and immunoblotted for total EGFR, phospho (p)-EGFR Tyr845, pEGFR Tyr1068, pEGFR Tyr1173 and pERK1/2. Densitometric analysis was performed and results were normalised to *β*-actin levels. The data illustrated are representative of three separate experiments. (**C**) After growing to 70% confluency and serum starvation for 24 h, cells were challenged with EGF (5 min). Samples were electrophoresed and immunoblotted for total EGFR, pEGFR Tyr1068, pEGFR Tyr1173, total Akt, pAkt, total ERK1/2 and pERK1/2 and analysed as detailed in (**B**). (**D**) Cells were harvested for RNA, RT–PCR was performed and the resulting cDNA was amplified using primer sets for TGF-*α*. Fragments were resolved on agarose gels and densitometric scores were normalised to *β*-actin. Data represents mean values of three experiments and error bars indicating 95% CIs.

When compared to the matching control, LoVo cells grown in medium containing 10% serum, showed a growth inhibition of 16.1% (CI=7.9–24.3, *P*=0.03) when challenged with 1 *μ*M gefitinib (Figure 1A). Cells grown in medium containing 7.5, 5, 2.5 1, 0.5 and 0% serum showed 25.7, 16.9, 8.9, 16.4, 18.1 and 27.6% growth inhibition to gefitinib, respectively, and in comparison with the growth inhibition determined in the presence of 10% serum, it was noted that reducing the serum content did not reveal a further significant growth response to the inhibitor (with *P*-values being 0.768 or greater) (Figure 1A).

It should be noted that throughout the study, following densitometric analysis and normalisation of the Western blot data, control values were taken to represent 100% and an average increase or decrease greater than 20% after experimental treatment from triplicate samples was considered to represent changes in expression or activity of the protein of interest.

The LoVo cells expressed considerable basal amounts of EGFR protein, which was phosphorylated (Figure 1B). The effect of gefitinib on the phosphorylation of specific tyrosine residues within the EGFR demonstrated that compared with the controls, the gefitinib-treated LoVo cells consistently showed a reduction in pEGFR Tyr845 activity, the tyrosine site located in the kinase domain of the receptor, whereas the levels of activity of the downstream autophosphorylation sites pEGFR Tyr1068 and pEGFR Tyr1173, however, appeared unaltered (Figure 1B). Similarly, gefitinib was without any apparent inhibitory effect on the activity of pERK1/2 (Figure 1B). In order to confirm that the EGFR signalling pathway was intact in these cells, it was established that challenge with EGF produced marked increases in the activity of pEGFR Tyr1068/1173 which was accompanied by increases in the activity of the downstream signalling targets Akt and ERK1/2 whereas the expression of EGFR, Akt and ERK1/2 remained unchanged (Figure 1C). It was also shown by RT–PCR studies that the LoVo cells produced considerable levels of the EGFR ligand TGF-*α* in comparison to A549 and DU145 cells (Figure 1D).

### Expression of components of the IGF-1R and InsR signalling pathway

**Figure 2:**
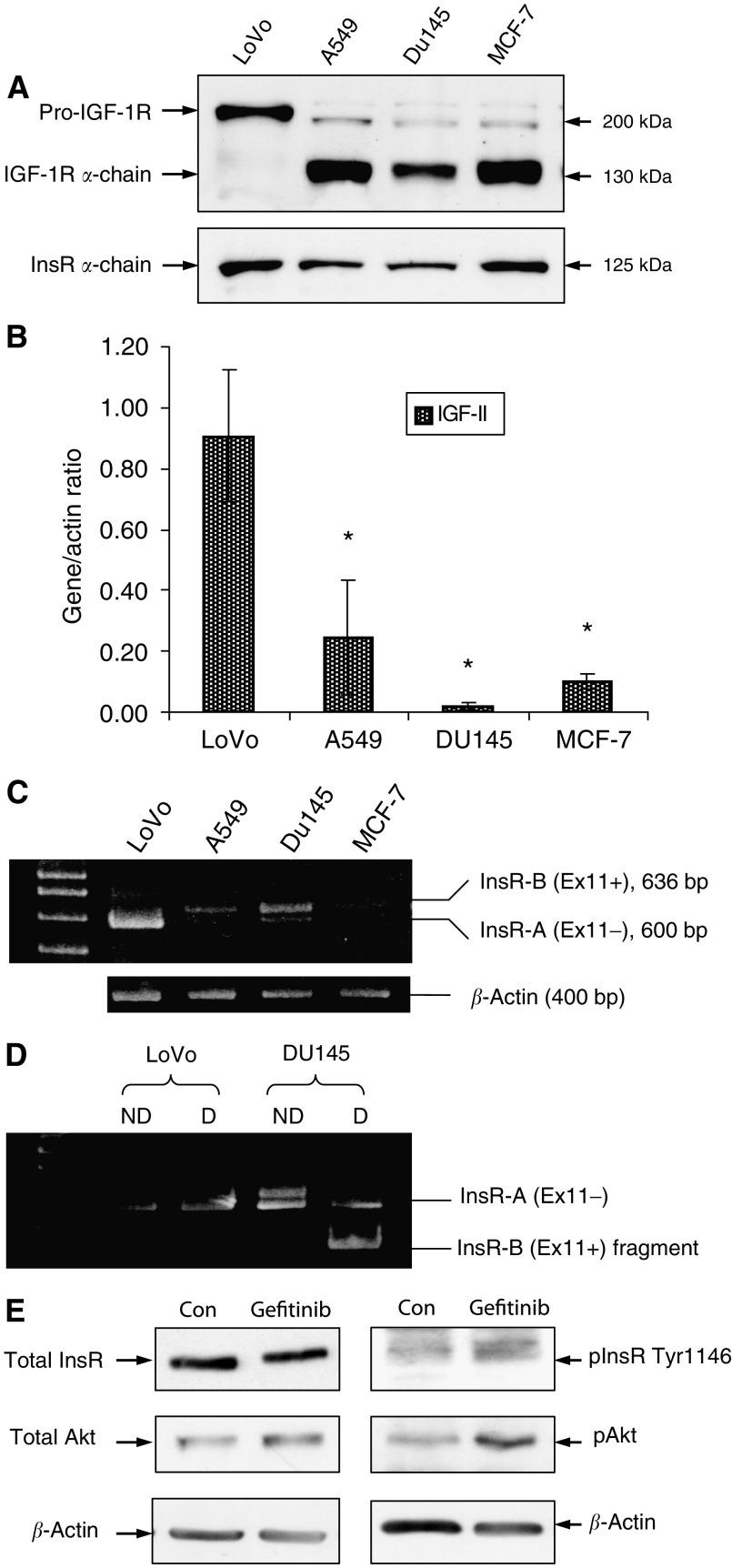
Evaluation of IGF-1R and InsR signalling pathway components. LoVo, A549, DU145 and MCF-7 cells were grown in DCCM-1 with 0.5% serum for 7 days. (**A**) Protein (75 *μ*g) of cell lysate was electrophoresed by SDS–PAGE (7.5%) and immunoblotted for total IGF-1R and InsR. (**B**) Cells were harvested for RNA, RT–PCR was performed and the resulting cDNA was amplified using primer sets for IGF-II. Fragments were resolved on agarose gels and densitometric scores were normalised to *β*-actin. Data represents mean values of three experiments and error bars indicating 95% CIs. ^*^Shows significant differences assessed between LoVo cells and each of the other cell lines at the *P*<0.05 level. (**C**) Amplified InsR-A and InsR-B cDNA was resolved by PAGE (15%) and normalised to *β*-actin. (**D**) Following PCR amplification for InsR-A and InsR-B, the PCR products were digested with *Ban I* restriction enzyme, before resolution by PAGE. Data illustrates nondigested (ND) and digested (D) cDNA fragments in LoVo and DU145 samples. (**E**) Cells were grown in DCCM-1 with 0.5% serum in the absence and presence of 1 *μ*M gefitinib for 7 days. Protein (75 *μ*g) of cell lysate was electrophoresed by SDS–PAGE (7.5%) and immunoblotted for total and phosphorylated InsR and Akt.

LoVo cells expressed high levels of uncleaved pro-IGF-1R protein, represented by the band seen at ∼200 kDa and no mature receptor at 130 kDa, in contrast to A549 (non-small-cell lung carcinoma), DU145 (prostate) and MCF-7 (breast) cancer cell lines (Figure 2A). The LoVo cells, however, produced mature InsR protein as determined by the presence of a band at 125 kDa (Figure 2A), whereas immature InsR at ∼200 kD was not detected in any of the cell lines evaluated. RT–PCR studies subsequently indicated that compared with the A549, DU145 and MCF-7 cells, the LoVo cells produced significantly higher (3.6–45-fold, *P*<0.001) levels of IGF-II mRNA (Figure 2B). Insulin mRNA was not detected in any of the cell lines.

The presence of the A and B isoforms of the InsR was assessed and RT–PCR studies showed the existence of 600 and 636 bp fragments, representing Ins-A (Ex11−) and Ins-B (Ex11+), respectively, and also indicated that in comparison with the A549, DU145 and MCF-7 cancer cells, LoVo cells consistently expressed elevated amounts of InsR-A mRNA, with minimal detection of InsR-B mRNA (Figure 2C). As exon 11 contains the restriction site for *Ban*I restriction enzyme, the presence of InsR-A (Ex11−) was confirmed by its resistance to degradation by the enzyme. For example, in the DU145 cells, only InsR-B containing Exon 11 is digested by *Ban1*, whereas the InsR-A in both LoVo and DU145 cells remained intact (Figure 2D).

Western blotting analysis further showed that the LoVo cells contained phosphorylated InsR which moreover, demonstrated a consistent increase in activity after challenge with gefitinib. InsR expression appeared to be unaltered after exposure to gefitinib (Figure 2E). In addition both the expression and activity of Akt, a common downstream target for InsR signalling, was elevated in all samples studied following exposure to gefitinib (Figure 2E).

### Effect of growth factors and the InsR/IGF-1R inhibitor ABDP on cell proliferation

**Figure 3:**
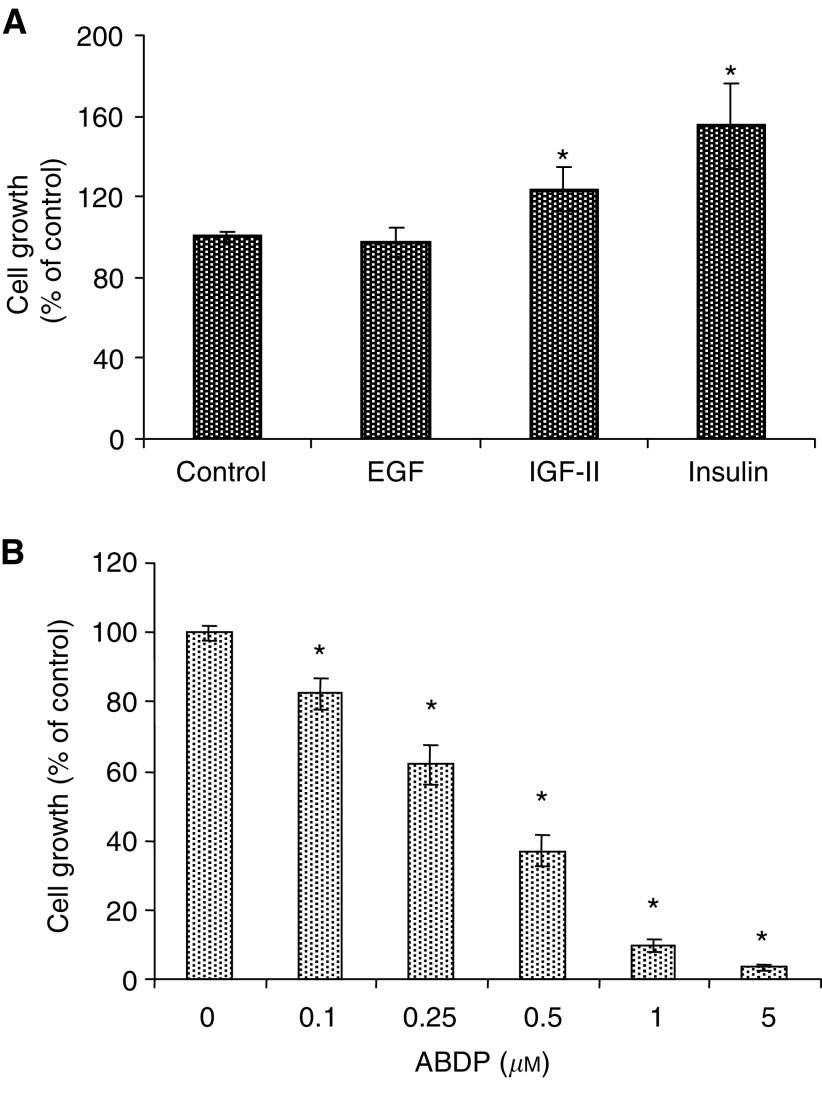
Growth responses of LoVo cells to mitogenic growth factors and the InsR/IGF-1R inhibitor ABDP. (**A**) LoVo cells were challenged with EGF, IGF-II (both at 10 ng ml^−1^) and insulin (10 *μ*g ml^−1^) for 7 days in DCCM-1 serum-free medium. Data are mean values of three independent experiments (every point in each individual experiment also being evaluated in triplicate) and error bars represent 95% CIs. ^*^Significant differences were assessed at the *P*<0.05 level. (**B**) LoVo cells were grown in the absence and presence of varying concentrations of ABDP (0–5 *μ*M) in DCCM-1 with 0.5% serum for 7 days. Data are mean values of three independent experiments (every point in each individual experiment also being evaluated in triplicate) and error bars represent 95% CIs. ^*^Significant differences were assessed at the *P*<0.05 level.

The potential importance of type II RTK signalling in mediating LoVo cell growth was demonstrated by the observation that small significant increases in growth were promoted by IGF-II (mean 24%, CI=13–35, *P*=0.025) and insulin (mean 55%, CI=34–76, *P*=0.009) compared with control values (Figure 3A). In contrast, EGF was without significant effect (mean 98%, CI=91–104, *P*=0.999).

Challenge of the cells with the InsR/IGF-1R inhibitor ABDP, revealed a role for InsR in the basal growth of the cells, where it resulted in significant (*P*<0.001) dose-dependent decreases in LoVo cell number (IC_50_=0.4 *μ*M) for example at 1 *μ*M ABDP, mean decrease 90.4%, CI=88.4–92.4, *P*<0.001 (Figure 3B). ABDP inhibits both IGF-1R and InsR tyrosine kinase activity, with increased selectivity for the IGF-1R. Given the observation that the InsR is the predominant mature type II RTK in LoVo cells, however, it is likely that any effects seen as a result of exposure to the inhibitor are exerted through the InsR.

### Modulation of InsR-A and EGFR phosphorylation by insulin, IGF and ABDP

**Figure 4:**
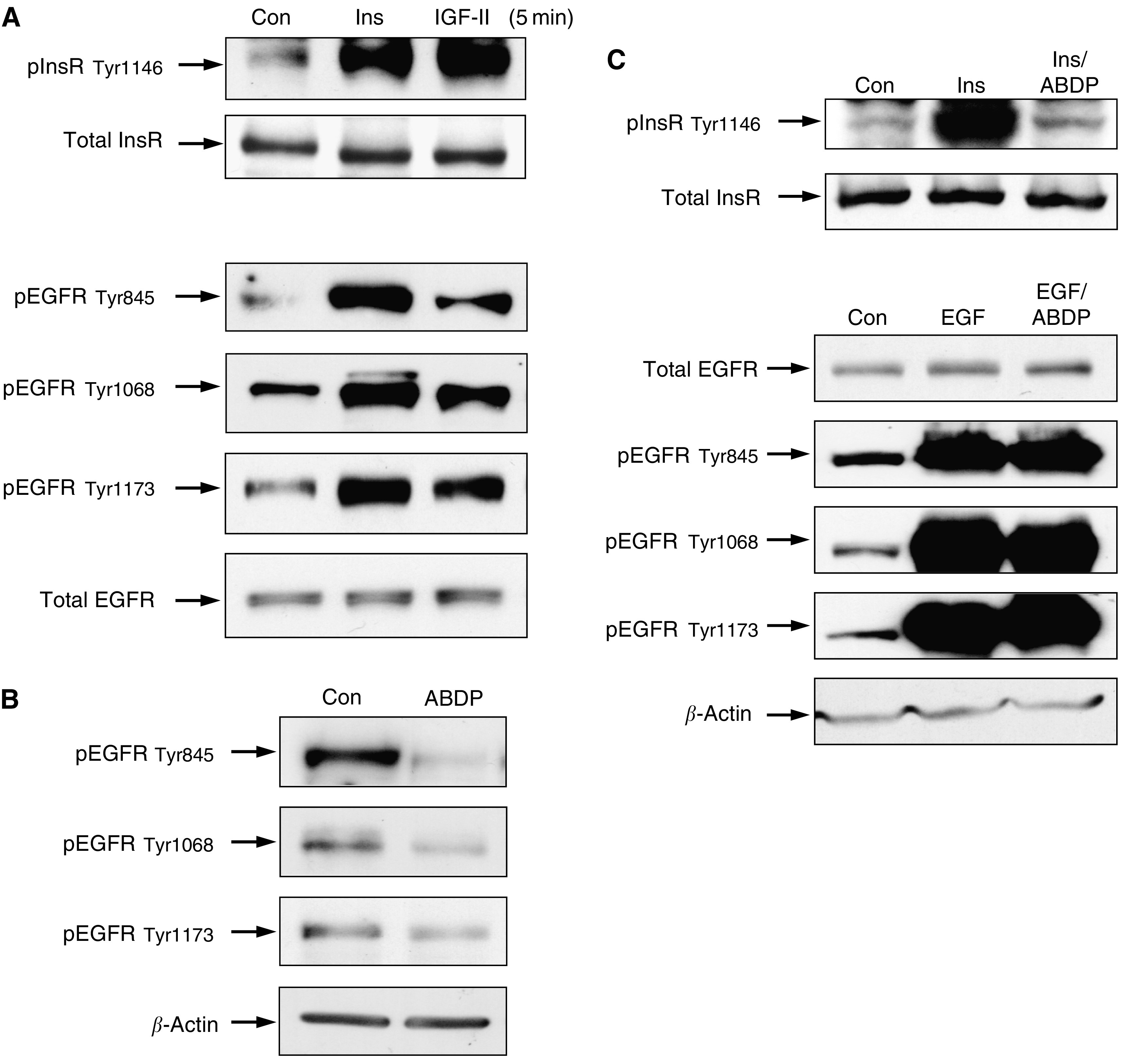
Modulation of pEGFR activity by insulin and IGF-II and the InsR/IGF-1R inhibitor ABDP. (**A**) LoVo cells were grown in routine culture medium until 70% confluent and after 24 h in serum-free DCCM-1 were challenged with IGF-II (10 ng ml^−1^) and insulin (10 *μ*g ml^−1^) for 5 min. Cell lysates (75 *μ*g) were electrophoresed by SDS–PAGE (7.5%) and immunoblotted for total InsR, pInsR Tyr1146, total EGFR, pEGFR Tyr845, pEGFR Tyr1068, pEGFR Tyr1173. Densitometric analysis was performed and results were normalised to *β*-actin levels. The data illustrated are representative of three separate experiments. (**B**) LoVo cells were grown in the absence and presence of 1 *μ*M ABDP in DCCM-1 with 0.5% serum for 7 days and electrophoresed, immunoblotted for pEGFR Tyr845, pEGFR Tyr1068, pEGFR Tyr1173 and analysed as described in (**A**). (**C**) LoVo cells were grown in routine culture medium until 70% confluent and after 24 h in DCCM-1, were challenged with insulin (10 *μ*g ml^−1^) and EGF (10 ng ml^−1^) with and without 1 *μ*M ABDP for 5 min. Cells exposed to ABDP were preincubated with this inhibitor for 6 h before electrophoresis, immunoblotting for total InsR, pInsR Tyr1146, pEGFR Tyr845, pEGFR Tyr1068, pEGFR Tyr1173 and analysis as described in (**A**).

Marked increases in pInsR were seen after challenge with insulin and IGF-II, while total InsR expression remained constant. (Figure 4A). This was complimented by consistent increases in pEGFR Tyr845, pEGFR Tyr1068 and pEGFR Tyr1173 following a 5 min stimulation with insulin and IGF-II while total EGFR expression appeared unaltered (Figure 4A). The effect of ABDP on EGFR phosphorylation indicated that in the presence of this inhibitor pEGFR Tyr 845, pEGFR Tyr1068 and pEGFR Tyr 1173 were all consistently reduced (Figure 4B).

The effectiveness and specificity of ABDP was confirmed as the inhibitor prevented the strong stimulation of InsR phosphorylation by insulin, however, EGF-induced phosphorylation of the EGFR at multiple tyrosine sites was unaffected by the inhibitor. The expression of both total InsR and EGFR appeared unaltered by the treatments (Figure 4C). Furthermore, it was observed that ABDP could prevent IGF-II and insulin-induced growth promotion in the LoVo cells, but as these cells do not respond to EGF, the specificity of ABDP could not be definitively demonstrated by anchorage-dependent growth assays (data not shown).

### Combination studies using gefitinib and ABDP

**Figure 5:**
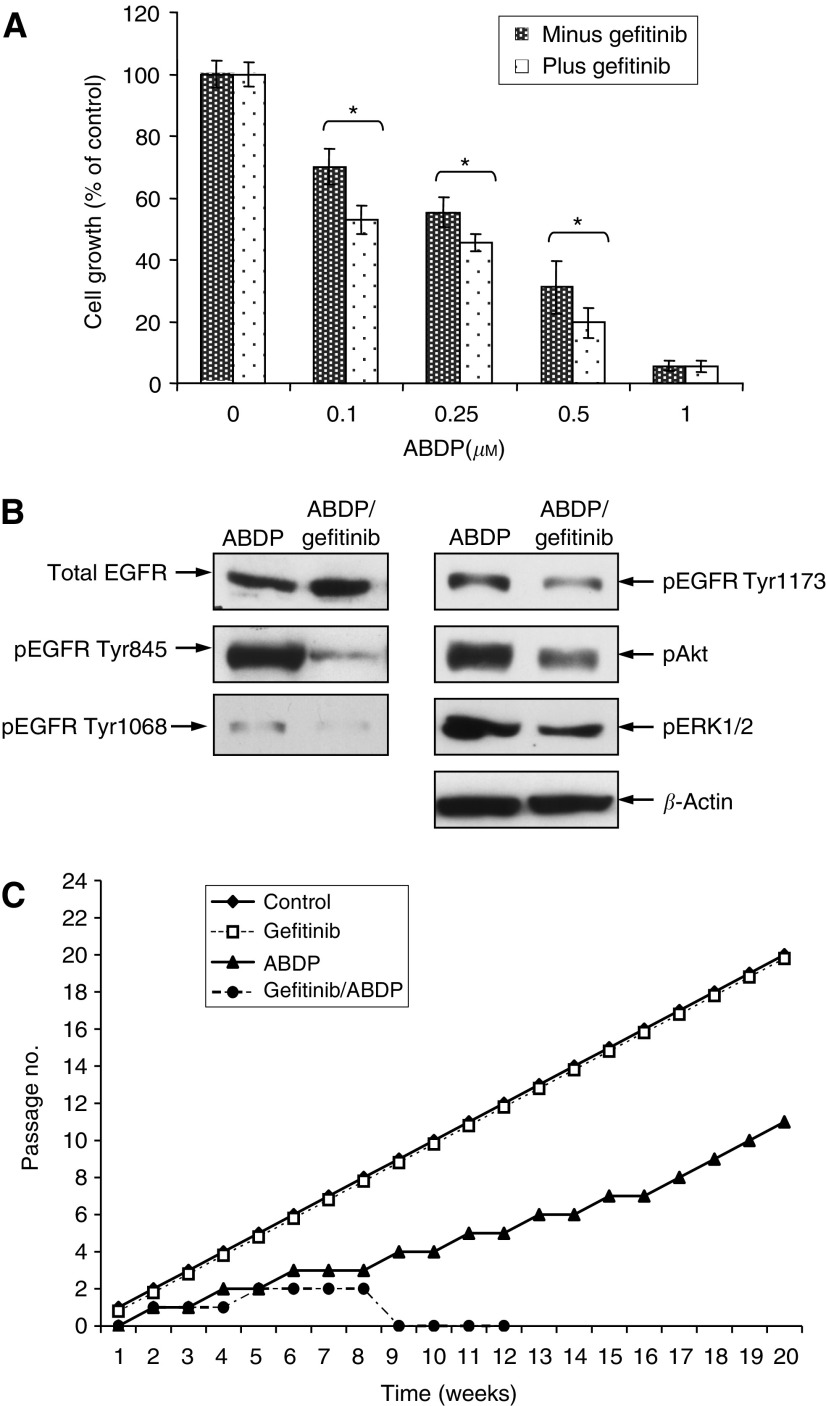
Gefitinib and ABDP in combination are more effective than either agent alone. (**A**) LoVo cells were cultured in varying concentrations of ABDP (0–1 *μ*M) in the absence (filled bar) or presence (spotted bar) of 1 *μ*M gefitinib in DCCM-1 with 0.5% serum for 7 days. Data are mean values of three independent experiments (every point in each individual experiment also being evaluated in triplicate) and error bars represent 95% CIs. ^*^Significant differences were assessed at the *P*<0.05 level. (**B**) LoVo cells were grown in DCCM-1 with 0.5% serum containing 1 *μ*M ABDP for 4 days and subsequently challenged with ABDP and gefitinib in combination for 24 h. Cell lysate (75 *μ*g) was electrophoresed by SDS–PAGE (7.5%) and immunoblotted for total EGFR, pEGFR Tyr845, pEGFR Tyr1068, pEGFR Tyr1173, pAkt and pERK1/2. Data was assessed by densitometry and are representative of three separate experiments. (**C**) LoVo cells were chronically exposed to control media i.e. DCCM-1 with 0.5% serum (-♦-), 1 *μ*M gefitinib (^…^□^…^), 1 *μ*M ABDP (-▴-) or gefitinib/ABDP dual treatment (-•-) until cell loss occurred or resistance developed. The data demonstrates the total passage number with respect to time (weeks).

The effect of gefitinib and ABDP in combination on cell proliferation *vs* ABDP alone was evaluated. It was demonstrated that the combination treatment produced small but significant additive effects on the inhibition of cell growth compared with ABDP as a single agent after short-term (7 days) challenge, that is, mean decrease 17.2%, CI=12.2–22.2, *P*<0.001 at 0.1 *μ*M ABDP, mean decrease 10.2%, CI=7.2–13.2, *P*<0.001 at 0.25 *μ*M ABDP and mean decrease 18%, CI=11–25, *P*<0.001 at 0.5 *μ*M ABDP (Figure 5A). Western blotting studies subsequently showed that when InsR activity was minimised, sensitivity to gefitinib was restored as following a period of prior incubation with ABDP, subsequent challenge with gefitinib resulted in a consistent reduction in pEGFR Tyr845, Tyr1068 and Tyr1173, which was complemented by a fall in both ERK1/2 and Akt activation (Figure 5B). These observations were in complete contrast to the data obtained when LoVo cells were challenged with gefitinib as a single agent, and such control samples were also undertaken within these experimental samples and the results were as already presented in Figures 1B and 2E, which shows that gefitinib has no effect on EGFR/ERK1/2 activity and increases Akt activity. Equally it was confirmed that ABDP reduced EGFR activity exactly as already shown in Figure 4B. The minimising of InsR signalling was demonstrated by the inability of insulin to stimulate InsR phosphorylation in the ABDP containing samples (data not shown but similar to that illustrated in Figure 4C). Importantly, the small additive effect on growth inhibition of the ABDP/gefitinib combination seen over the short-term in Figure 5A, however, translated out into total cell loss over chronic exposure after approximately 9 weeks. Moreover, LoVo cells continually treated with ABDP alone, although initially growth inhibited, eventually developed resistance and the stable resistant subline (LoVo-ABDP-R) was established by 20 weeks (Figure 5C). No difference was noted between control cell growth and gefitinib-treated cells over long-term challenge (Figure 5C).

### Characterisation of LoVo-ABDP-R cell line

**Figure 6:**
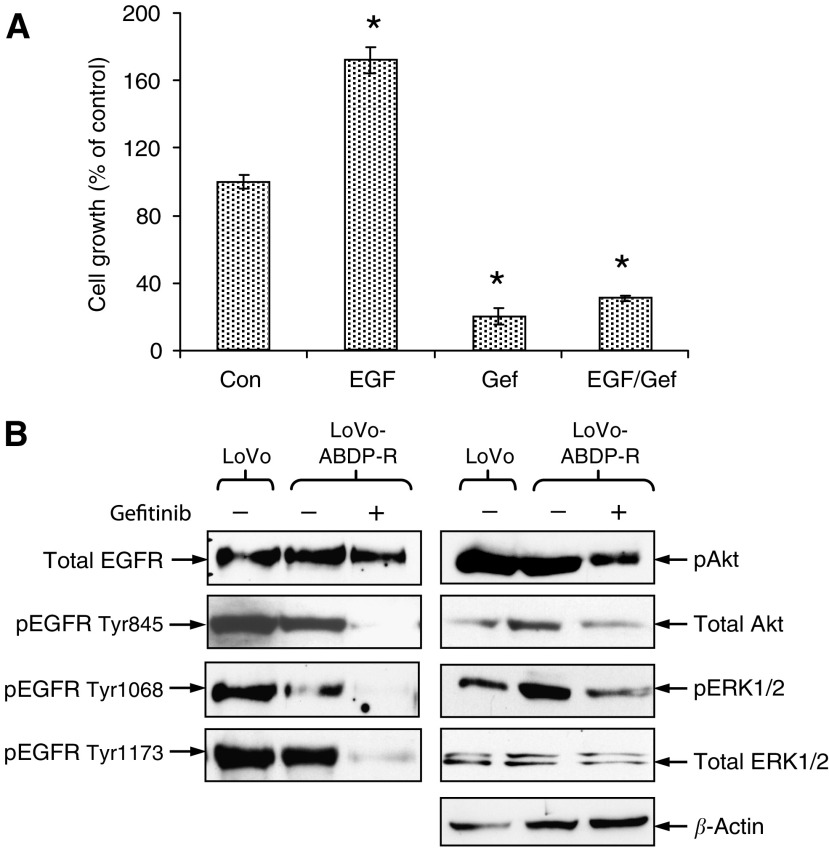
LoVo-ABDP-R cells have gained sensitivity to gefitinib. (**A**) LoVo-ABDP-R cells were challenged with EGF (10 ng ml^−1^), gefitinib (1 *μ*M) and EGF/gefitinib together for 7 days in DCCM-1 serum-free medium containing 1 *μ*M ABDP. Data are mean values of three independent experiments (every point in each individual experiment also being evaluated in triplicate) and error bars represent 95% CIs. ^*^Significant differences were assessed at the *P*<0.05 level. (**B**) LoVo cells were grown in DCCM-1 with 0.5% serum for 7 days and LoVo-ABDP-R cells were cultured in the absence and presence of 1 *μ*M gefitinib for 7 days in DCCM-1 supplemented with 0.5% serum and 1 *μ*M ABDP for 7 days. Protein (75 *μ*g) of cell lysate was electrophoresed by SDS–PAGE (7.5%) and immunoblotted for total EGFR, pEGFR Tyr845, pEGFR Tyr1068, pEGFR Tyr1173, total ERK1/2, pERK1/2, total Akt and pAkt. Densitometric analysis was performed and results were normalised to total expression levels. The data illustrated are representative of three separate experiments.

The effect of EGF and gefitinib the growth of the LoVo-ABDP-R cell line was determined. Significant increases in growth were promoted by EGF (mean 72%, CI=65–79, *P*<0.001) compared with control values (Figure 6A). Gefitinib treatment resulted in a dramatic reduction (mean 79%, CI=75–83, *P*<0.001) in basal cell growth and prevented EGF-induced growth (mean 69%, CI=67–71, *P*<0.001) (Figure 6A). Although ABDP reduced the considerable basal pEGFR Tyr845, Tyr1068 and Tyr1173 activity in the LoVo cells (Figure 4B), interestingly, the LoVo-ABDP-R cells showed similar basal levels of EGFR phosphorylation to their untreated parents indicating a substantial recovery of EGFR activity (Figure 6B) which moreover, could be consistently effectively reduced by gefitinib (Figure 6B). This was accompanied by a concomitant fall in the activity of both ERK1/2 and Akt (Figure 6B). Hence acquisition of resistance to ABDP has created a response to gefitinib (Figure 6B). In addition, compared to the parental LoVo cell line, the ABDP-resistant variant demonstrated similar levels of expression of InsR, which showed minimal stimulation after challenge with insulin or IGF-II (data not shown).

## DISCUSSION

Drug resistance, either *de novo* or acquired after initial response, to molecular-targeted anticancer drugs such as gefitinib is an emerging clinical problem (Vidal *et al*, 2004) as we seek to fulfil their therapeutic potential. Indeed, LoVo CRC cells have abundant EGFR expression and activity, yet show only modest sensitivity to gefitinib as a single agent, despite the inhibitor being used at a concentration previously shown to be highly efficacious in other EGFR-positive cell lines (Jones *et al*, 2004). We have previously shown that increased IGF-1R signalling plays a key role in the development of acquired resistance to gefitinib in breast and prostate cancer cells (Jones *et al*, 2004) and we have postulated that such signalling, if present at high levels in cancer cells, may be responsible for *de novo* resistance to EGFR blockade. Interestingly, however, although the LoVo cells were able to produce pro-IGF-1R, they were unable to process it to a mature phosphorylated species and this receptor therefore, is unlikely to play a role in the *de novo* gefitinib resistance seen in these cells. This conclusion is further supported by studies from other workers which demonstrated that in LoVo cells, this pro-IGF-1R cannot transduce intracellular signals (Lehmann *et al*, 1998). The inability of the LoVo cells to produce mature IGF-1R is consistent with their deficiency in the proprotein convertase furin, which is required to generate the mature or active forms of numerous latent proteins including the IGF-1R (Lehmann *et al*, 1998) and the InsR (Robertson *et al*, 1993). Surprisingly, however, mature phosphorylated InsR was present in the cells suggesting that the InsR was processed via an alternative furin-independent pathway. It has been shown, for example, that although cleavage by furin is regarded as a key event in the activation of membrane type-1 metalloproteinase (MT1-MMP), in furin-devoid LoVo cells, MT1-MMP could rapidly be converted into its activated form via an alternative route (Deryugina *et al*, 2004). Interestingly, RT-PCR analysis showed that the LoVo cells preferentially expressed InsR-A isoform as opposed to InsR-B and also produced substantial levels of IGF-II mRNA, a known ligand for InsR-A (Frasca *et al*, 1999; Sciacca *et al*, 1999). This suggests that the activity of the InsR-A may be promoted in an autocrine fashion, a concept confirmed by our findings that not only insulin but also IGF-II, significantly induced cell proliferation and conversely, substantial growth inhibition was seen after challenge with the IGF-1R/InsR inhibitor ABDP. Indeed, differential expression of the InsR-A by cancer cells compared to their normal counterparts has been reported in colon, lung, breast (Frasca *et al*, 1999) and thyroid cancer clinical samples (Vella *et al*, 2002) and additionally, these cancer types frequently overexpress IGF-II (Quinn *et al*, 1996; Renehan *et al*, 2000). In addition, other studies have shown that whereas IGF-II mainly induces mitogenesis after activating the InsR-A, insulin can also promote cell proliferation after binding to the InsR-A, as well as being able to drive metabolic events via this InsR isoform (Frasca *et al*, 1999).

Significantly, exposure of the LoVo cells to insulin and IGF-II, not only promoted the activation of InsR-A signalling but also increased EGFR phosphorylation at residues Tyr845, Tyr1068 and Tyr1173, whose activation moreover, could be substantially reduced after exposure to the InsR/IGF-1R inhibitor ABDP. In comparison, gefitinib had no effect on pEGFR Tyr1068 and Tyr1173 and only a partial inhibitory effect on pEGFR Tyr845 activity. This lack of effect of gefitinib on EGFR activity was not due to receptor dysfunction as EGF challenge substantially increased EGFR activity, together with Akt and ERK1/2 activity indicating that the EGFR signalling pathway was intact. On the basis of all these data, we hypothesised that the lack of growth inhibitory effects of gefitinib in LoVo cells was due to the activation of the EGFR by InsR-A signalling, Furthermore, since gefitinib treatment also increased InsR-A signalling and downstream Akt activity, it is possible that gefitinib acts to limit its own efficacy, essentially aiding the promotion of the transactivation of the EGFR by the InsR-A. It is established that modulation of EGFR activity can occur by several routes including crosstalk or transactivation by other erbB family members (Gullick, 2001) but critically, by other heterologous growth factor receptors such as the IGF-1R (reviewed by Adams *et al*, 2004) and we have shown that the InsR-A, which shares a high degree of structural homology with the IGF-1R (De Meyts and Wittaker, 2002), may also engage in crosstalk with the EGFR. We have previously demonstrated that in our tamoxifen resistant breast cancer cells, the IGF-1R can modulate pEGFR Tyr845 activity via mechanism dependent upon the non receptor tyrosine kinase c-Src (Knowlden *et al*, 2004) and furthermore, other studies have shown that in colon cancer cell lines with high IGF-1R expression, the IGF-1R can activate c-Src to modify cell transformation and motility (Sekharam *et al*, 2003). Consequently, the role of c-Src as a potential intermediate between the InsR and EGFR is being evaluated in the LoVo cells.

From these current results, we would predict that blockade of InsR-A signalling might improve the efficacy of gefitinib and indeed, our studies showed that a combinatorial strategy of gefitinib and ABDP to simultaneously target the EGFR and the InsR-A, respectively, demonstrated that over 7 days, a small but significant additive effect on growth inhibition with the dual treatment was seen compared with ABDP alone. Excitingly, however, chronic exposure to ABDP revealed that while the cells were able to overcome its effectiveness and develop resistance to this inhibitor, the apparent initial small effect of the gefitinib/ABDP combination translated out into a substantial increase in cell loss preventing the development of acquired resistance to ABDP. Signalling analysis showed that whereas alone, gefitinib had no effect on the activity of the EGFR, under conditions where InsR-A signalling was blocked, gefitinib effects were restored as the inhibitor could reduce pEGFR at the multiple tyrosine residues and subsequently a decrease in ERK1/2 and Akt activation was also noted. Interestingly, other workers have shown that the anti-HER-2 agent traztuzumab could only inhibit the growth of MCF-7/HER-18 cells, which over-express HER-2 receptors and express IGF-1R, when IGF-1R signalling was minimised (Lu *et al*, 2001). Critically our results show that gefitinib, although having little effect on LoVo cell growth as a monotherapy, is a crucial component in a combination strategy that additionally targets the InsR, as a dynamic interplay exists between the EGFR and the InsR. Such observations are further exemplified by our findings that the LoVo cells which have gained acquired resistance to the InsR inhibitor ABDP, show a reliance on EGFR signalling as demonstrated by their growth promotion by EGF, recovered levels of EGFR phosphorylation compared with their ABDP-treated parents and extreme sensitivity to growth inhibition by gefitinib exerted via a fall in EGFR activity, each observation being in complete contrast to those shown by the parental cells.

In summary, the conceptual ideal where drugs specifically target elements involved in cancer, providing greater activity and specificity than is currently offered by traditional chemotherapeutic agents has proved to have some obstacles. Function redundancy, signalling via alternative signalling pathways or growth factor receptor crosstalk has shown that the antitumour activity of monotherapeutic regimes are easily subverted. Our results have extended our previous observations of the importance of type II RTKs in gefitinib resistance and support the rationale of combination strategies that target the InsR-A or IGF-1R in concert with the EGFR for maximum antitumour effects. It is also noteworthy that the capability of the InsR-A to crosstalk with the EGFR has important implications for the use of highly specific IGF-1R inhibitors which are currently being developed and evaluated (Hofmann and Garcia-Echeverria, 2005) and may be used as a monotherapy or in cotargeting strategies with EGFR inhibitors, as in this scenario, the resulting uninhibited InsR-A could conceivably substitute for the IGF-1R providing a potential resistance mechanism to IGF-1R inhibition.
